# The socioeconomic burden of pelvic floor disorders

**DOI:** 10.1111/aogs.70264

**Published:** 2026-05-19

**Authors:** Ian Milsom, Ida E. K. Nilsson, Jwan Al‐Mukhtar Othman, Jennie Larsudd‐Kåverud, Adrian Wagg, Maria Gyhagen

**Affiliations:** ^1^ Gothenburg Continence Research Centre Institute of Clinical Sciences, Sahlgrenska Academy at Gothenburg University Gothenburg Sweden; ^2^ Department of Obstetrics and Gynecology Sahlgrenska University Hospital Gothenburg Sweden; ^3^ Department of Obstetrics and Gynecology Södra Älvsborg Hospital Borås Sweden; ^4^ Department of Medicine University of Alberta Edmonton Alberta Canada

**Keywords:** fecal incontinence, obstetric anal sphincter injury, pelvic floor dysfunction, pelvic organ prolapse, urinary incontinence, vaginal delivery

## Abstract

Pelvic floor disorders (PFDs) such as pelvic organ prolapse (POP), urinary incontinence (UI), and fecal incontinence (FI) affect millions of women throughout the world. The aim of this review was to describe the socioeconomic burden of PFDs in women. A comprehensive literature search was performed in relevant databases to identify articles on this topic. The prevalence of pelvic floor disorders reaches up to 46% in adult women and many have >1 PFD. PFDs impair a woman's well‐being, quality of life, and sexual function and prevent many women from participating in recreational and sporting activities. UI alone constitutes a major health problem affecting the lives of more than 500 million persons worldwide. Combined FI and UI have been reported in 10% of women living in the community, increasing to almost 50% in nursing home residents. The prevalence of symptomatic pelvic organ prolapse in women has been reported to be between 4% and 11%. The costs of PFDs to health care systems and society are enormous, and approximately one in five women will undergo surgery for genital prolapse or UI by the age of 85 years. Global demographic trends regarding the aging population indicate that the prevalence of both UI and FI will rise in the coming years, significantly increasing the health and societal burden as well as the economic costs for both patients and health service payer. At present, PFDs incur a huge socioeconomic burden on women throughout the world. Management strategies during delivery should be individualized to reduce the burden of pelvic floor dysfunction currently occurring following vaginal birth.

## Graphical abstract

Pelvic floor disorders can have a negative influence on a woman's well‐being, quality of life, working ability, and sexual function and also prevent many women from participating in recreational activities. The global costs of PFDs to health care systems and society are enormous.

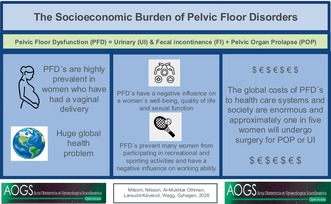

**Abbreviations**
FI: fecal incontinence
FSD: female sexual dysfunction
OASI: obstetric anal sphincter injury
PFD: pelvic floor disorder
POP: pelvic organ prolapse
QALYs: quality‐adjusted life‐years
QoL: quality of life
UI: urinary incontinence
VD: vaginal delivery

**Key message**

Pelvic floor disorders impose a substantial socioeconomic burden on women worldwide. Implementing individualized management strategies during childbirth may help reduce the incidence and long‐term impact of pelvic floor dysfunction following vaginal delivery.

## 1 INTRODUCTION

Pelvic floor disorders (PFDs) such as pelvic organ prolapse (POP), urinary incontinence (UI), and fecal incontinence (FI) constitute a huge global health problem affecting millions of women throughout the world.^1^ The prevalence of pelvic floor disorders has been reported to be 46%, and many women have >1 PFD.^1^ PFDs can have a negative influence on a woman's well‐being, quality of life, and sexual function and prevent many women from participating in recreational and sporting activities. The global costs of PFDs to health care systems and society are enormous^2^, ^3^, ^4^, ^5^ and approximately one in five women will undergo surgery for genital prolapse or UI by the age of 85 years.^6^

UI alone constitutes a major health problem affecting the lives of more than 500 million persons worldwide.^1^, ^7^, ^8^ The prevalence of UI increases with age and is highly prevalent in older women and those with cognitive impairment.^1^ FI occurs in up to 6% of those younger than 40 years, increasing to 15% in older individuals.^9^, ^10^, ^11^ Combined FI and UI have been reported in 10% of women living in the community, increasing to almost 50% in older nursing home residents.^12^, ^13^ Incontinence is also more prevalent in women living with neurological disease, for example, multiple sclerosis, spina bifida, Parkinson's disease, and stroke.^1^ Global demographic trends, especially increased life expectancy, will lead to an increase in the prevalence of both UI and FI, resulting in a greater health and social burden as well as an increased economic cost for both patients and health service payers.^1^ The prevalence of POP in women has been reported to be between 4% and 11%.^14^, ^15^, ^16^, ^17^ POP is a rare condition in nulliparous women and in women after one or several cesarean sections, indicating that the mode of delivery is more important than pregnancy alone.^18^, ^19^

The aim of this review was to describe the socioeconomic burden of PFDs in women.

## 2 MATERIAL AND METHODS

This review evaluated the socioeconomic consequences of POP, urinary and fecal incontinence (FI) in women based on currently available information in the literature. A comprehensive literature search was performed in November 2025 among relevant databases (Ovid MEDLINE, Ovid Embase, CINAHL, Scopus, Web of Science Core Collection, Cochrane Library (via Wiley) and ProQuest Dissertations & Theses) to identify articles on this topic. The digital search was complemented with a review by the authors of the 7th Edition of Incontinence^1^ in order to identify important contributions to the literature. The book Incontinence, the result of the International Consultation on Incontinence, is updated every third year and is written by a large number of leading international experts within the field.

### 2.1 Definitions

The following definitions were used:

UI was defined as an involuntary loss of urine according to the International Continence Society/International Urogynecological Association terminology. This profile included people experiencing stress urinary incontinence (SUI), urgency urinary incontinence (UUI), mixed urinary incontinence (MUI), and UI not covered by the other categories.^20^

FI was defined as loss of control of liquid or solid stool.^20^

Pelvic organ prolapse was defined according to International Continence Society/International Urogynecological Association terminology.^20^ Symptomatic POP was defined by the affirmation of the symptom “feeling a bulge” or affirmation of the combination of four other pelvic floor symptoms: “vaginal pain/discomfort” (often), “worsening upon heavy lifting” (yes), “need for manual reduction of the anterior vaginal wall” (often/sometimes/infrequently), and “urge urinary incontinence” (often/sometimes/infrequently), which might also lead to a symptomatic POP classification.^21^

### 2.2 Urinary incontinence

UI is a highly prevalent condition with a profound influence on well‐being and quality of life,^22^ as well as being of immense economic importance for health service provision.^1^, ^2^, ^3^, ^4^ Millions of women throughout the world are afflicted,^2^, ^3^ and there has been a growing interest in these symptoms due to increased awareness of the human and social implications for the individual sufferer. Population‐based studies have reported that UI is more common in women than men and that approximately 10% of all adult women suffer from UI.^2^, ^3^ Prevalence increases with increasing age, and in women aged >70 years, more than 30% of the female population is affected. Prevalence is even higher among the oldest old (80+) and among older nursing home residents.^1^, ^23^

Involuntary leakage of urine is perceived by many women, but is not always reported to a healthcare provider. However, an increasing awareness of the problem has, in recent years, attracted more patients to seek advice. In older women UI has variably been reported as an important factor in the decision whether or not to institutionalize an older person.^2^, ^24^ Urinary incontinence not only causes personal suffering for the individual afflicted but is also of considerable economic importance for the health service.^2^, ^4^ The annual cost of UI in Sweden, for example, has been reported to account for approximately 2% of the total healthcare budget.^1^, ^2^

### 2.3 Pelvic organ prolapse

Pelvic organ prolapse refers to the loss of support for the uterus, bladder, colon or rectum leading to descent of one or more of these organs into the vagina.^1^ In addition to mechanical discomfort, POP may negatively affect sexuality, body image and quality of life. Globally up to half of all parous women have some degree of clinical prolapse and 10–20% are symptomatic.^1^, ^6^, ^25^, ^26^, ^27^ POP is one of the most common reasons for gynecologic surgery peaking in upper midlife.^1^, ^6^, ^25^, ^26^, ^27^ The lifetime risk of POP surgery has been reported to be 11–19% in welfare states.^6^, ^25^, ^26^, ^27^

### 2.4 Fecal incontinence

Fecal incontinence is a particularly distressing form of PFD with far‐reaching consequences affecting women's physical, psychological, and social well‐being. Involuntary bowel leakage is associated with stigma leading to social withdrawal, reduced self‐esteem, impaired sexual function, and a diminished quality of life.^1^, ^28^, ^29^ Despite its profound impact, FI is underdiagnosed and undertreated, largely due to feelings of shame, the normalization of symptoms, and a lack of awareness, particularly among older women. With rising life expectancy and more women remaining professionally active later in life, the societal impact is becoming increasingly evident.^30^, ^31^ FI occurs in 6% of those younger than 40 years, increasing to 15% in older individuals.^9^, ^10^, ^11^ Combined FI and UI have been reported in 10% of women living in the community, increasing to almost 50% in nursing home residents.^12^, ^13^

## 3 THE IMPACT OF PELVIC FOOR DYSFUNCTION ON QUALITY OF LIFE AND WORKING ABILITY

Urinary incontinence can impact many aspects of women's lives including physical exercise, work productivity, sleep, social interaction, travel, and sexual health. Women with UI consistently report having a lower quality of life (QoL) compared with those who are continent. A range of physical and psychosocial issues influence how UI affects QoL. The type and severity of UI, age, and concurrent medical conditions are important considerations but individual personality and coping strategies, social support, and culture also affect women's perception and management of the condition.^32^, ^33^, ^34^

Incontinence can directly or indirectly have a negative impact on women's ability to participate in paid employment. Those affected by the condition may not be able to work due to suboptimal incontinence management while others providing care to a family member with incontinence may not have time to take on additional work.^35^

Pelvic organ prolapse can cause pelvic, urinary, bowel, and sexual symptoms, leading to impaired quality of life and working ability. Jobs involving heavy lifting have been reported to increase the risk for POP. In a multicenter study, Woodman et al^36^ showed that the prevalence of POP was higher among women who were laborers or factory workers. They also reported that an annual household income of 10 000 US$ or less was associated with severe POP.^36^

## 4 THE IMPACT OF PELVIC FLOOR DYSFUNCTION ON SEXUAL FUNCTION

It has been estimated that 50–60% of women with PFD are sexually active.^37^, ^38^ Data regarding the presence of dyspareunia, decreased orgasmic capacity, and libido in women with POP are controversial. Studies have demonstrated that symptomatic POP and UI increase the risk of female sexual dysfunction (FSD) due to reduced arousal, infrequent orgasm, and dyspareunia, while women with UI are more likely to avoid sexual intimacy due to their fear of urinary leakage.^39^, ^40^ However, Solhaug et al demonstrated that the negative impact of incontinence on sexual life was less prevalent 10–20 years after sling surgery compared with the preoperative assessment.^41^ FSD is related to the presence of POP.^42^ The negative effect of POP on sexual function may be improved or may remain unchanged following PFD surgery.^43^

Fecal incontinence also significantly impairs sexual health, leading to lower sexual satisfaction, desire, and function due to a fear of leaks, odor, pain, and other physical and emotional factors. While FI does not necessarily prevent sexual activity, individuals with FI report more difficulties with lubrication, pain, and orgasm.^44^ Managing the condition through treatment, open communication with a partner, and proactive measures like pre‐sexual cleansing, emptying the bladder, and using protective products can help improve sexual well‐being.^44^

## 5 THE ECONOMIC CONSEQUENCES OF PELVIC FLOOR DYSFUNCTION

In addition to the impact on health and well‐being, urinary and FI and POP impose a considerable cost to individuals and families, health services, and society in general.^3^, ^4^, ^5^, ^45^, ^46^, ^47^ The magnitude of these costs is not precisely known, but the annual costs of UI in both men and women in the United States were estimated to be over US$26.2 billion in 1995.^48^, ^49^ The economic consequences of UI in Sweden were assessed by Ekelund et al in 1990. The estimated annual cost for UI in Sweden at that time was 1.8 billion Swedish crowns. The Swedish Health Care budget for 1990 amounted to 93 billion Swedish crowns. Based on the results of this evaluation, the annual costs of UI in Sweden accounted for approximately 2% of the total healthcare costs. Ten years later, the annual estimated costs had increased from 1.8 billion Swedish crowns to 2.8–4.4 billion Swedish crowns.^50^ These costs will have increased further due to an aging population and price inflation. Villoro et al estimated that, in Spain alone, more than 350 000 Quality‐adjusted life‐years (QALYs) were lost due to UI among women aged ≥60 years. Valuing each QALY at US$50000, this equates to an estimated societal cost of approximately US$17.5 billion (2019 US$).^51^ With respect to overactive bladder associated with UI, a cost analysis in 2007 estimated a total annual direct health care cost of US$65.9 billion, with a projected cost of US$76.2 billion in 2015 and US$82.6 billion in 2020 for overactive bladder associated with UI.^52^

In older women, UI is a risk factor for social isolation and physical deconditioning; it is associated with depression, falls, early mortality, and impairs QoL. It increases the risk of institutionalization for older adults in the community and has an immense economic burden for healthcare systems, which is largely generated by costs for the care of older people with incontinence.^2^, ^3^, ^23^, ^53^, ^54^, ^55^ Data for the cost of UI in older adults (all, aged 65+) suggest that in the United States in 1995, $26.3 billion, or $3565 per individual with UI was consumed.^49^ For nursing home residents, this cost is increased by the associated costs of labor for care, and in 2003 was estimated at an additional $4957 annually per resident.^56^ The estimated cost of UI‐related complications in older adults in Canada has recently been assessed and was found to be a substantial economic burden to the Canadian Health Care System.^57^

Fecal incontinence has significant social, psychological, and economic ramifications.^9^, ^58^, ^59^, ^60^ It compromises QoL for patients as well as having a significant concomitant impact on patients' families, caregivers, friends, and the healthcare team. As the general population grows, the prevalence of FI will result in a substantial increase in the economic burden both on patients and society.^60^, ^61^, ^62^ The full economic cost of FI is not clear with few new data available in the last 20 years. A 1995 estimate of community‐based healthcare costs for FI exceeded US$11 billion annually in the United States.^62^ If adjusted for the rate of inflation, the comparative 2021 annual rate of healthcare cost for FI would exceed $19.1 billion per year.^3^ Sung et al. estimated that inpatient procedures for female FI alone cost $24.5 million (in 2003 US dollars). There are few, if any, data on the costs of conservative management for affected women.^47^, ^62^

Data on the economic burden of genitourinary prolapse are few but costs appear to be rising, given that treatment seeking and hospital activity related to care is on the rise. A study using 1997 National Hospital Discharge Survey data on volume and Medicare reimbursement rates for physician services and hospitalizations estimated the cost of prolapse surgery in the United States at $1.012 billion. It has been estimated that in the United States, roughly 9.2 million women will be affected by POP by 2050,^62^ with approximately 300 000 undergoing surgery each year at a cost of over 1 billion US$. Similar estimates from Europe were provided by Subramanian et al, who estimated that the annual cost of managing POP in 2005 Euros was €144 m, €83 m, and €81 m in Germany, France, and England, respectively.^63^

These costs illustrate that considerable economic resources are allocated to managing PFDs.

## 6 HOW CAN WE REDUCE THE SOCIOECONOMIC BURDEN OF PELVIC FLOOR DISORDERS IN THE FUTURE?

There is a wealth of evidence demonstrating the socioeconomic burden of pelvic floor disorders. The question, therefore, arises: Can we prevent PFDs in the future based on currently available knowledge?^64^ The etiology of PFDs is multifactorial, and numerous risk factors have been identified, some of which can be prevented.^1^ Pregnancy and in particular obstetric trauma during vaginal childbirth are important risk factors for UI, FI, and POP. Vaginal birth, in particular, instrumental vaginal birth is the most important factor in the etiology of UI, FI, and POP.^1^, ^65^

The scientific literature clearly demonstrates the importance of vaginal delivery (VD) in the development of UI and FI, and the occurrence of obstetrical anal sphincter rupture (OASI) is of particular importance for FI.^1^, ^65^ Obstetricians and midwives should identify women at risk, provide antenatal counseling and plan appropriate management on how to prevent urinary and FI and other pelvic floor dysfunctions. In Norway, the implementation of perineal protection programs, delivered as a bundle of care consisting of several coordinated components, has been successful in preventing OASI.^66^, ^67^ The use of a lateral episiotomy in nulliparous women requiring vacuum extraction during delivery has also been shown to reduce the risk of OASI.^68^ The presence of two assisting midwives at late second stage of labor has also been shown to prevent severe perineal trauma.^69^ Numerous risk factors associated with VD have been identified, including maternal height and body mass index, neonatal birth weight, and instrumental delivery.^1^ These factors have enabled the development of scoring systems and predictive models to support obstetricians and midwives in the management of an individual woman's forthcoming delivery. The UR‐choice model was developed in order to provide mothers to be with information about the risk of future pelvic floor dysfunction.^70^ The concept put forward in the UR‐choice model was evaluated further in a prediction model based on two large population‐based studies with long‐term results regarding the risk of developing pelvic floor dysfunction.^71^ More recently, a study has been published regarding the development and validation of a prediction model to identify women at risk for OASI.^72^

Our knowledge regarding the etiology of UI and FI can be used to individualize management of pregnant women and their delivery. Individual management during delivery, taking into account relevant factors that may influence future pelvic floor dysfunction, may reduce the burden of pelvic floor dysfunction currently occurring following vaginal birth.

## AUTHOR CONTRIBUTIONS

All authors contributed to the conceptualization and preparation of this literature review. The final version of this manuscript was approved by all authors.

## FUNDING INFORMATION

The study was financed by grants from the Swedish state under the agreement between the Swedish Government and the county councils, the ALF agreement under grant number ALFGBG‐966115 (Milsom), ALFGBG‐933810 (Gyhagen), Hjalmar Svensson's Fund under grant number HJSV2022018. The Health & Medical Care Committee of the Region Västra Götaland, VGFOUREG‐967583. The funding sources had no role in the study design, data analysis, and interpretation, or report writing.

## CONFLCIT OF INTEREST STATEMENT

M.G. reports receiving honoraria from Essity Hygiene & Healthcare, Sweden, and Astellas Pharma. I.M. reports receiving honoraria for lectures from Essity Hygiene & Healthcare, Astellas Pharma, Pfizer, Pierre Fabre Laboratories, and Allergan. AW reports receiving honoraria from Astellas Pharma and Becton‐Dickinson, research support from Essity Hygiene and Healthcare and Becton‐Dickinson. The remaining authors report no conflict of interest.

## Data Availability

The data that support the findings of this study are available from the corresponding author upon reasonable request.
